# Supplementing Program Profiles in Evidence Clearinghouses with Insights for Practice: a Qualitative Investigation of Application to Youth Mentoring Programs in CrimeSolutions

**DOI:** 10.1007/s11121-025-01841-8

**Published:** 2025-10-08

**Authors:** Aisha N. Griffith, Julia Pryce, David L. DuBois, Timothy Brezina, Kelly E. Stewart, Michael Garringer

**Affiliations:** 1 Department of Educational Psychology, University of Illinois Chicago, Chicago, IL USA; 2 School of Social Work, Loyola University Chicago, Chicago, IL USA; 3 Institute for Health Research and Policy and Division of Community Health Sciences, School of Public Health, University of Illinois Chicago, Chicago, IL USA; 4 Department of Criminal Justice & Criminology, Georgia State University, Atlanta, GA USA; 5 Institute for Health Research and Policy, School of Public Health, University of Illinois Chicago, Chicago, IL USA; 6 MENTOR, Boston, MA USA

**Keywords:** Youth mentoring programs, Program design, Evidence-based repositories, CrimeSolutions, Program implementation

## Abstract

Evidence-based program repositories have been designed to help practitioners in their decision-making. Most repositories supplement summaries of effectiveness evidence with information intended to assist with implementation of each included program (e.g., training costs). It is less common for guidance to be included to support translation of findings for a broader range of purposes, such as enhancing related programs already in place. To help address this gap within the area of youth mentoring, the National Mentoring Resource Center has appended “Insights for Mentoring Practitioners” to profiles of 47 mentoring programs included in the CrimeSolutions.gov repository of the National Institute of Justice. We qualitatively analyzed these commentaries to elucidate themes across them that can inform the development and improvement of mentoring programs. Themes included (1) ensuring alignment across program goals, design, implementation, and evaluation; (2) connecting the intervention to mentees’ home, parents, and larger environment; (3) tailoring mentor engagement and support to effectively serve youth; and (4) optimizing the role of mentoring within multi-component programs. Discussion focuses on how findings inform the improvement of mentoring programs, and how content geared toward the translation of evidence to practice could enhance and improve evidence repositories.

**Supplementary Information:**

The online version contains supplementary material available at 10.1007/s11121-025-01841-8.

## Introduction

One of the most popular approaches to strengthen the research foundations of applied interventions is to identify those with evaluations that meet a clear and demanding scientific standard for assessing effectiveness, and catalog information about the programs and their evidence base into a publicly accessible clearinghouse. Examples include *Blueprints for Healthy Youth Development*, the *What Works Clearinghouse* of the Institute for Education Sciences, *CrimeSolutions* of the National Institute of Justice (NIJ), and the *Results First Clearinghouse* Database, developed by the Pew Charitable Trusts, which consolidates information on the effectiveness of social policy programs provided in the foregoing repositories, along with six others. In essence, these repositories seek to address the question, “What works?,” based on a standardized system of assessing the rigor and substance of the evidence on the effectiveness of different programs or interventions, typically as obtained in experimental or well-designed quasi-experimental evaluations. Developed with practitioners and policymakers in mind, these repositories provide easy-to-digest ratings for each program evaluated. For example, CrimeSolutions rates programs as “effective,” “promising,” or “no effects.” This study focuses specifically on CrimeSolutions’ application of ratings to youth mentoring programs.

Despite the value of such repositories in disseminating key information, they address a fairly narrow research question. As Feucht and Tyson (2018) observe, the question of what works, by itself, can overlook the complexity of program evaluations and the contextual factors that influence evaluation outcomes:

Rather than framing evaluation results only in terms of “what works” (or what does not work), we might address other important questions more completely. Under what circumstances did the program work and not work? What aspects of the program contributed to its effectiveness or lack thereof?... Knowledge is cumulative, time and place are dynamic, and research is iterative. Pretending otherwise limits the value of what we learn from any single study (p. 177).

Consideration of qualitative data has been argued to be valuable in addressing limitations that arise in the process of translating findings to practice (Titler, 2004). Qualitative data sourced from non-researchers, such as research-to-practice intermediaries, may be of particular value, especially when used for hypothesis-generation (e.g., theory development) rather than more definitive hypothesis-testing (Glynn, 2021).

Furthermore, in the absence of additional context and guidance, program effectiveness ratings may be of limited value to end users of evidence-based repositories. Theoretically, the programs in these evidence-based repositories are picked up and utilized. But many users will be interested in enhancing existing programs rather than the wholesale adoption of new programs. Indeed, in focus groups with users of evidence-based repositories, Stephenson et al. (2014) identified “adaptability” as a key theme, noting that while program fidelity was seen as important, the ability to adapt or modify existing programs emerged as a priority among participants. Moreover, even if users are in the market for a new program, selecting one will likely entail considerations regarding one’s local context. These include the extent to which a program addresses locally identified needs, fits with local values and resources, and leverages community strengths and assets. There is little information on what should guide decision-making in this area (Feucht & Tyson, 2018). This is particularly important in mentoring, as adult mentors often come from different communities and backgrounds than mentees. Thus, ideally, programs are preparing mentors to be attuned to the local community and youth strengths, rather than implementing a program that assumes mentoring relationships can be established without this consideration.

A subtle limitation is that research on programs found to be ineffective may nonetheless include useful implications for practice. Such programs can reveal pitfalls to avoid or conditions that may mitigate the impact of otherwise well-designed efforts. Additionally, what failed to work under certain conditions may work in other contexts. Yet, little of this type of information is in current repositories, such that contents are limited to factual information. Feucht and Tyson (2018) note, “It’s likely that most CrimeSolutions.gov users readily disregard programs rated 'no effects,' perhaps assuming these programs contain nothing of value” (p. 177). To foster the productive utilization of repository information, they endorse formative efforts taken by evidence-based repositories to provide more contextual information and draw out practical implications through literature reviews and implementation guides. They stress that “these important developments need to be supported and extended further” (p. 180).

### National Mentoring Resource Center’s (NMRC) Insights for Mentoring Practitioners

In line with this call, the NMRC, established in 2014 as a program of the Office of Juvenile Justice and Delinquency Prevention (OJJDP), has produced Insights for Mentoring Practitioners (hereafter “Insights”) to accompany the profiles of mentoring programs for youth that are included in NIJ’s CrimeSolutions repository, whether or not these programs were rated as effective or not. The stated purpose of this supplemental information is *“*to give mentoring professionals additional information and understanding that can help them apply reviews to their own programs.” More specifically, the Insights for a given program typically highlight between four and six points for reflection or “takeaways.” Most of these observations revolve around features of the program’s goals, design, implementation, and other programmatic considerations, such as the backgrounds of participating mentors and youth, that may be important in considering its demonstrated effectiveness (or lack thereof) and the ways in which practitioners could potentially apply these to their own programs. Each commentary is prepared by the Senior Director of Research and Quality for MENTOR, the organization that has been funded continuously to implement the NMRC since its inception. See details on the process for creating these commentaries in the “Methods” section. The Director of Research for the NMRC reviews each Insight to ensure accurate and appropriately contextualized content.

The NMRC contributes the commentaries as a way to add value to the standard profiles used to describe each program that is included in CrimeSolutions and its evidence base with respect to effectiveness. Each profile includes a description of the program (e.g., goals, primary components, staffing requirements), a detailed summary of the methodology used in up to three experimental (randomized control) or quasi-experimental evaluation(s) that have passed initial screening for minimum rigor and represent the evidence base for the program’s effectiveness, and findings with respect to overall effects of the program on justice-related outcomes, such as delinquency and related problem behaviors and associated risk and protective factors. Information pertinent to program implementation (e.g., cost, availability of a program manual) and selected other results from the evaluations (e.g., findings of tests for possible subgroup differences in program effects) also may be included when available. Although the quantitative evaluations that the CrimeSolutions repository uses have many strengths, they have limited ability to shed light on *process* or “mechanisms by which a particular intervention instigates a series of events that ultimately result in the observed impact” (Rao & Woolcock, 2003). The NMRC believes the Insights could speak to processes in a way that practitioners could apply.

Their usefulness may be limited, however, for several reasons. First, one may overgeneralize from Insights derived from a single program. Second, it is inefficient to read through all the available Insights. Third, even if it were possible to consume all the information, emergent themes across the Practitioner Insights may be overlooked. To address these concerns, this study uses qualitative methods to synthesize information contained in Insights. The analysis sought to identify themes in the commentaries in the context of also considering program information and effectiveness ratings provided in the CrimeSolutions profiles of the programs.

Our approach reflects growing recognition that while quantitative findings on program effectiveness have great value, they often miss less easily measured and quantified, contextual factors that can shape program effectiveness in a given instance (e.g., particular program, population, set of outcomes, etc.), as well as miss identifying the translational implications of findings with respect to application to similar but not identical programs, populations, and so forth (see DuBois, 2017, for such arguments). Approaches like Community-Based Participatory Research and Participatory Team Science emphasize the essential role of non-researcher stakeholders in co-producing and interpreting findings and their potential applications (Collins et al. 2018; Tebes, 2018). From an epistemological standpoint, these approaches align with a shift from strict logical empiricism to contextualism and perspectivism in community and prevention science (Tebes, 2005). Recommendations flowing from perspectivism include: 1) viewing all research-generated knowledge as yielding only an approximation of the “truth,” such that community and prevention science should emphasize hypothesis-generation, not only hypothesis-testing, as a means of advancing knowledge and should rely on multiple methods to obtain the best approximation of the “truth,” and 2) that “to be applicable to a diverse array of people and settings, community science should balance its focus on internal validity with one that also emphasizes external and ecological validity” (Tebes, 2005, p. 222). While often advanced with respect to individual studies, parallel arguments have been put forth regarding efforts to synthesize evidence from multiple studies (see, e.g., Pluye & Hong, 2014, for discussion of mixed methods reviews based on the premise that combining qualitative and quantitative approaches to evidence synthesis compensates for their respective limitations).

### Why Focus on Youth Mentoring Programs?

Several considerations support the usefulness of undertaking this analysis with a focus on mentoring programs. First, mentoring is one of the most widely utilized approaches to promote positive development and prevent the emergence of adaptive difficulties among youth, particularly those from economically disadvantaged backgrounds (DuBois & Karcher, 2014), and is estimated to serve at least 2.5 million youth each year (Garringer et al., 2017). Yet, mentoring programs have a history of being developed in large part at the local level, outside of a research context. Whereas the programs in CrimeSolutions are often created by researchers testing an intervention, mentoring programs frequently are developed and implemented in response to prioritized areas of need for young persons within a given community and, by their nature, are oriented toward capitalizing on their internal resources through reliance on community members as mentors (DuBois & Karcher, 2014). Such circumstances make it likely that practitioners will not be well-positioned to utilize programs that have been developed and received rigorous evaluation in other contexts as is, as they apply evidence to inform more localized programmatic efforts. Thus, Insights serve as a tool to transfer knowledge from research that can be leveraged locally.

Although there is a robust base of evidence to indicate that the youth in mentoring programs often benefit in areas like problem behavior avoidance and relationships with parents and peers (e.g., Raposa et al., 2019; Tolan et al., 2014), the effects tend to be relatively small and vary across and within programs. Mentoring programs could strengthen their theories of change (Garringer, 2014) by drawing on relevant parts of evaluations of other, sometimes very different, programs, which may help them consider what they do and processes by which they can do this better at their local level. Importantly, programs need to be responsive to the unique needs of youth of color, who comprise the majority of participants in most programs (Hagler et al., 2023). These considerations highlight the need for guidance on how to apply evidence on program effectiveness in more nuanced ways. Finally, the mechanisms by which a mentoring program is implemented at the program level in general can shape the impact of the intervention.

## Method

### Background on CrimeSolutions

We drew data from the CrimeSolutions website and the NMRC’s Program Reviews website, which included programs that:

- were evaluated in the Model Programs Guide of the Office of Juvenile Justice and Delinquency Prevention between January 2014 and January 2022;
- were reviewed through the CrimeSolutions review process;
- provided sufficient research evidence to assign an effectiveness rating;
- maintained a Program Profile Information on the CrimeSolutions website;
- included a Practitioner Insight for Mentoring Practitioners on the NMRC website.

This yielded review data of 47 mentoring programs. Studies constituting the evidence base for these programs were published between 2002 and 2021 and implemented experimental or quasi-experimental research designs. While most (*n* = 34) had a randomized controlled trial as part of their evidence base, just over half (*n* = 24) had been evaluated with a high-quality RCT study as defined by CrimeSolutions,^1^ and 5 were evaluated across multiple samples. Program studies and outcomes relating to juvenile justice or the prevention of justice system involvement were selected for inclusion in CrimeSolutions using a standardized process. A majority (61.7%) targeted at least one academic or career-related outcome such as GPA (*n* = 11), attendance (*n* = *7*), graduation (*n* = 11), failure or dropout risk (*n* = 3), attending college (*n* = 6), or career development and employment (*n* = 5); slightly more than half (53.2%) targeted a social-emotional learning (SEL; *n* = 16) or mental health-related outcome (*n* = 14), and just under half (40.4%) targeted at least one justice-related outcome, such as arrests/offending (*n* = 8). Our analysis focuses on the Insights; however, quantitative analyses were also conducted to test for possible differences in program characteristics of those rated promising or effective (*n* = 30) relative to those rated “no effects” (*n* = 17). No statistically significant differences emerged between the groups in program characteristics measured, including the presence of non-mentoring components (e.g., structured curricula delivered by non-mentors), program setting (i.e., rural communities versus urban and suburban only), delivery setting (i.e., school- versus community-based), format (i.e., exclusively one-to-one versus a group component), mentor age (i.e., adult versus older peers), mentees’ age (i.e., exclusively children under 12, pre-teens ages 12–14, teens ages 13 and above, or a variety of ages), race/ethnic identity of youth served (i.e., Hispanic, Black, white, Asian/Pacific Islanders, American Indian/Alaska Native youth), and diversity of race/ethnic identity of youth served (i.e., sum of the number of race/ethnic identity categories programs reported serving). This finding aligns with meta-analytic studies which, with few exceptions, have found that basic program characteristics, like mentoring format, have little bearing on the potential to have a positive impact on youth (DuBois et al., 2002; Raposa et al., 2019).

### Data Sample

The Supplementary Material has examples of the Insights. Each 2–4 page narrative provides the link to the review on the CrimeSolutions.gov website and its effectiveness rating. It then provides contextualizing information related to practice and highlights key takeaways, practices that might have influenced a program’s rating, design considerations, and implementation tips aimed at translating information to practitioners. Insights were developed prior to this study’s analysis.

Written by the Senior Director of Research and Quality at MENTOR, the author of these Insights is well-positioned to provide commentary on each study of a program, with an aim toward translating research-based content for practitioners. The Director utilized his nearly two decades of experience supporting practitioners with program implementation through technical assistance, authoring and editing commonly used guidebooks (e.g., the 4th edition of the *Elements of Effective Practice for Mentoring)*, and developing tools for programs. These experiences shaped a philosophical orientation that one can often learn more by looking at the seeds of a program's failure than simply admiring and copying a program rated as effective that might never be perfectly replicated. As such, Insights were written to help developers consider what works, why it works, and under what conditions, so developers do not unintentionally implement programs that are rated as effective but may be a poor fit for their own context. Thus, Insights about programs that were rated “no effects” were written in as much depth as those rated effective. While there is no standardized process for determining commentary content, the Senior Director considers several questions to identify “key takeaways” for practice: What implementation challenges may have negatively influenced the quality of mentoring relationships or the effectiveness of the program? Were there any novel or innovative aspects of the program design or delivery that seemed to enhance program effectiveness? Do any of the findings from the evaluation build on or contradict the findings of other research studies on mentoring? Are there lingering questions or unknown factors due to the methodology of the evaluation that may obscure the implications of a finding? The content of each commentary builds on these questions to help practitioners consider elements of the program design and delivery that they may want to emulate in their own programs, as well as potential pitfalls and challenges to avoid in the planning and implementation of their programs. The commentaries also highlight evaluation methodologies programs may want to consider for future use.

### Data Analysis

Insights were analyzed qualitatively by the first two authors to identify themes (i.e., recurring or related takeaway points) across the Insights written for each program. Both analysts have expertise in youth-adult relationships and qualitative research; neither has an affiliation with the reviewed studies or programs. A multi-step process was used for analysis, which was initially guided by conventional content analysis (Hseih & Shannon, 2005), followed by thematic analysis based on themes (Braun & Clarke, 2006). First, the two researchers divided the Insights equally. Each researcher then undertook an initial review of the content of their assigned Insights by program group (i.e., no effects, promising, effective), focusing on familiarizing themselves with the data with particular attention to facilitators and barriers to effectiveness. While reading, each researcher created a table with notes and interpretations, as well as a set of concepts reflected in the data (e.g., “challenges to rigorous fidelity in implementation” or “Interesting in terms of youth characteristics, this program failed in addressing significantly challenged youth, but another addressed this with intensive mentor training.”). These notes were compiled in a shared Google Doc to inform their early analytic meetings. During meetings, researchers reviewed each other’s notes to discuss overlap in their notes around key concepts, interpretations, and supporting data.

This process generated an extensive set of key concepts, which were then discussed by the two researchers in terms of shared interpretation and areas of discrepancy. In subsequent meetings and reviews of the Insights, identified concepts were increasingly organized into iterative codes and clusters of meaning (Braun & Clarke, 2006). Throughout, the researchers consulted reflexively with several members of the larger team, who were familiar with the profiles and the process by which the Insights were developed, to clarify language and format regarding themes (Padgett, 2006). These team members approached the data from various epistemological orientations, as well as levels of familiarity with the data, which added rigor to the analysis. Additional strategies for ensuring rigor included building confirmability by documenting analytical decisions and peer debriefing (Padgett, 2006). At the conclusion of this process, the majority of the most salient concepts were grouped into themes, drawn from original content, and outlined here. (A less salient concept around “cost and impact” will be referenced in the “Discussion” section rather than the Results because of how slim it was in the data.) This article focuses on findings from research completed in connection with the NMRC. A report was made available on the NMRC website as required by the funder. This is an updated version of part of this report. The work (in current or earlier form) has not been published in a journal.

## Results

Programs varied greatly in terms of the range of youth served, geographic location, community settings, and format for mentoring. While most programs took place in schools or community settings, there were also examples of mentoring in the home, on college campuses, and in the workplace, as well as some instances of e-mentoring. Mentoring mostly occurred one-to-one and was delivered by an adult, but about a third of programs included group mentoring, and about a fifth included peer mentoring. A summary of the programs and evaluations in this review, organized by evidence rating, can be found in the Supplementary Material.

Analyses led to four core themes across Insights: (1) ensuring alignment across goals, design, implementation, and evaluation; (2) connecting the intervention to mentees’ home, parents, and larger environment; (3) tailoring mentor engagement and support to effectively serve youth; and (4) optimizing the role of mentoring within multi-component programs. These themes suggest factors that may contribute to program effectiveness that could not be captured through quantitative analysis or without a nuanced look at the Insights commentaries.

### Ensuring Alignment Across Goals, Design, Implementation, and Evaluation

The most prominent theme was the importance of ensuring that a program’s design, implementation, and evaluation are in alignment, both with one another and with the program’s goals as reflected in program aims. Insights suggested that programs rated as effective (*n* = 3) and promising (*n* = 27) reflected intentionality in their development, deliberately focused on aligning practices with program and evaluation characteristics (e.g., theory, training, fidelity, outcomes evaluated). Programs rated as no effects (*n* = 17) typically lacked this intentionality. Multi-faceted alignment or misalignment emerged in various ways.

At times, alignment involved coherence between theory and implementation, built on a consideration of the characteristics of the youth served. In the case of Better Futures, for example, the Insights argued that program designers selected self-determination theory to guide their mentoring efforts because they served foster care youth “who, by the nature of their experience in the child welfare system, may have felt disempowered in the course of their life paths.” Thus, Better Futures was built on the idea that youth should feel empowered to take an active role in their postsecondary planning. This aim was achieved by providing youth with “just enough mentoring, skill development, and instrumental support” to place them in the “driver’s seat.”

In other instances, multi-faceted alignment was reflected by coherence between program theory and implementation to achieve fidelity. Quantum Opportunities, an effective program serving 9th graders through graduation, provides a duration and depth of mentoring, alongside other supports and services, that fits with its theory of change. In addition to providing long-term mentoring, the program clearly delineated the role of mentors and “sought to determine the ideal number of hours that a student would participate in mentoring, tutoring, leadership training, and the other program activities over the course of a year.” This example highlights how mentoring is approached intentionally within effective programs. Program components are carefully considered and monitored, alongside other components, with program goals in mind.

While Quantum Opportunities provides long-term mentoring, other mentoring programs rated as effective or promising are relatively short in duration. The question of program duration is therefore complicated. As suggested by the Insights, program efficacy may have more to do with intentional alignment than with mentoring duration per se. Regarding the Youth Advocate Program (YAP), the Insights note that the relatively short duration of this promising mentoring program may seem surprising because it serves youth involved in the justice system for serious and violent offenses. Yet, what YAP lacks in duration, “it more than makes up for in intensity.” The Insights outline YAP’s intensive and flexible model and incorporation of paid mentors, who work from 7.5–30 h per week, depending on a mentee’s presenting needs. Therefore, although it is a short-term program, it remains intentional in terms of fit and flexibility between mentee needs and program delivery, and therefore demonstrates promise as assessed by these Insights.

Multi-faceted alignment also involves aligning program expectations with those of the mentors. This coherence strengthens fidelity to the program model. In the case of the National Guard Youth ChalleNGe program, the Insights suggest the rating of no effects was due to a lack of alignment between mentors’ expectations and program implementation in terms of a lack of clarity regarding the mentors’ role. The Insights state that a “takeaway for practitioners is that if mentors are to be used in supporting other intervention work, their role must be clearly defined and implemented with fidelity if it’s to be as effective as hoped.” It went on to state: “Mixed findings indicate that a program might be on the right path but could also take a fresh look at its theory of change and strengthen services in some key areas,” indicating an opportunity to “strengthen their model” by increasing alignment between implementation and mentor expectations.

Using continuous improvement to increase alignment was also alluded to in the Insights. In the case of Quantum Opportunities, for example, the Insights point out that this effective program “built on a much earlier iteration of the Quantum program” and “put a lot of thought into the communities where this program might be a good fit.” The Insights also state that, over time, and with the help of training and technical assistance, the program was able to adjust implementation across sites to meet local circumstances and service delivery needs. This fact allowed the program to thrive in different communities and produce consistent cross-site results.

As these examples illustrate, the alignment of program elements with real-world practice may be key to effectiveness. But the intentional alignment of program elements may also represent one of the greater challenges in program development and implementation. As stated in the Insights, the “real conundrum mentoring programs face is how they can both build on and implement research-based ‘effective’ practices and program models, while also allowing for enough flexibility to customize an intervention or specific practice for local context or needs.”

In short, when considering the facilitators and barriers associated with program efficacy, the intentional alignment of program elements emerged as a prominent theme in the Insights. For a summary of this and other core themes, along with related observations, see Table 1.

**Table 1:** Insights for mentoring practitioners: Core themes and related findings regarding facilitators and barriers associated with program efficacy

Core theme	Key findings associated with theme
Theme 1. Ensuring alignment across goals, design, implementation, and evaluation	Effective and Promising programs tend to be intentional in their development, aligning practices with program characteristics and intended outcomes. Intentional alignment tends to be multifaceted and is reflected in a good fit between the overall theory of change and the intensity, duration, and depth of mentoring; between mentee needs and program delivery; and between other program elements. In “aligned” programs, components are carefully considered at the design stage and continuously monitored during implementation, with program goals in mind.
Theme 2. Connecting the intervention to mentees’ home, parents, and larger environment	Effective and Promising programs tend to draw on a network of relational support (from parents, peers, teachers, or others) to support the role of the mentor. In programs reflecting this theme, mentors are not alone in supporting youth, but join others in doing so, and leverage the strength of these relationships to increase support for the mentee.
Theme 3. Tailoring mentor engagement and support to effectively serve youth	Effective and Promising programs tend to focus on the careful matching of mentors and youth based on relevant criteria and specific youth needs. Such programs carefully consider the role and tasks of mentors, and tailor recruitment, selection, preparation, and support to these roles and tasks
Theme 4. Optimizing the role of mentoring within multi-component programs	When programs include multiple components, those that are Effective and Promising tend to have mentoring components that are clearly delineated and easily identified. Too many program components can be counterproductive, potentially diluting the effects of mentoring, especially when the components are not fully aligned and integrated with each other. Within an evaluation framework, in multi-component programs, the contribution of mentoring to observed effects tends to be difficult to discern in the absence of research designs that are tailored to achieve this aim.

### Connecting the Intervention to Mentees’ Home, Parents, and Larger Environment

As an extension of the focus on intentionality in program design, other themes emerged that help illuminate the connection between the program and community resources. The second theme underscored the importance of connecting the home, family, and larger environment to support the impact of mentoring. Effective and promising programs were noted for intentionally engaging parents, peers, clinicians, or other members of the community to bolster the mentoring process. In such programs, mentors are not alone in supporting youth, but they join others in doing so, and leverage the strength of these relationships to increase support for the mentee.

For instance, Baloo and You, rated as promising, connects mentors with parents to support academic success. The Insights notes the program intentionally engages parents, stating: “mentors spend time with the parents and caregivers of mentees and, if the grades are coming around, really encourage them to have their child apply to the high track. This is likely something that many of these parents may have never even considered for their child. And into their life comes a college student who seems to be making a difference with their child and encouraging them to take the ‘path not taken.’”

In the case of Quantum Opportunities, rated as effective, the Insights state, “Mentors are expected to get to know the Associate’s [mentee’s] family and friends and integrate themselves into the existing web of support in the student’s life and community.” In this example, the program builds in expectations that mentors build relationships with mentees’ family and friends, thereby leveraging the strength of these relationships. The Sources of Strength program, rated as promising, engages peer leaders in the students’ school ecology to advance suicide prevention, and thereby leverages the power of cliques to disseminate information important to suicide prevention. This innovation expands the web of mentoring, while also illustrating alignment between very difficult and important program content (i.e., suicide risk and prevention) and youth access (i.e., through peer leaders as mentors), to create a promising program.

Other programs support effectiveness by engaging an even wider range of people in the mentee’s ecology, including teachers, social workers, and other professionals. Fostering Healthy Futures, a promising program, is an example of mentoring in partnership with mental health professionals. In this program, clinicians offer separate but complementary support to youth. It provides youth in foster care with a “blend of one-to-one mentoring and more direct clinical support, in this case a series of manualized and clinician-led skill-building group activities over 30 weeks.” As clinicians lead group training sessions for youth in foster care, mentors support mentees outside the groups. The Insights state, “this kind of focused clinician-led skills group training in addition to mentoring allows for mentors to focus on, well, mentoring.” In other words, engaging clinicians allows mentors to do what they do best, in this case supporting and enjoying their mentees, while trusting that trained professionals can more directly address mental health and well-being. Other promising and effective programs also demonstrate the potential value of intentionally incorporating other supports, and in some cases, professionals, to accompany the work of mentors, and ultimately strengthen the mentee outcomes of interest.

### Tailoring Mentor Engagement and Support to Effectively Serve Youth

Programs rated as effective and promising do not leave the mentoring relationship up to happenstance, and instead focus on the careful matching of mentors and youth based on relevant criteria and specific youth needs. These programs were noted for carefully considering the role and tasks of mentors, and they tailor recruitment, selection, preparation, and support to these roles and tasks. In doing so, these programs help mentors intentionally fulfill their role.

In terms of recruitment and selection, some Insights discussed how programs targeting a specific population were deliberate in choosing mentors to serve this population. Better Futures, rated as effective, provides an example. The Insights argue that a “factor in the success of Better Futures may be who they ask to fill the mentor role. The mentors in this program are all young adults who have been to college and who also themselves have been in the foster care system or dealt with mental health issues.” My Life, rated as promising, provides another example. This program carefully considered the coach/mentor role, recruiting mentors who were slightly older than mentees and “had been in foster care or wrestled with a disability themselves.”

The Insights indicate that supporting mentors selected is critical. Great Life Mentoring, rated as effective, serves youth who face mental health challenges. The program helps mentors fulfill their role by providing monthly supervision and tailored training aligned with the needs of such youth. Training covers topics deemed important for successfully mentoring youth served, such as attachment theory, self-awareness, emotional health, and healthy boundaries. As indicated in the Insights, this robust training, in combination with regular in-person supervision, “allows mentors to further learn about and act in support of the youth’s overall treatment plan and areas of emphasis for growth and change set out by the youth’s mental health providers.”

In contrast, Arches, a credible messenger program connecting youth to mentors with similar life experiences and rated as no effects, is described as largely missing their target due to a lack of support for mentors. The Insights stated that “the design of Arches seems tremendous on paper.” However, although “credible messengers brought tremendous skills and relatability to the role,” it was "clear that credible messengers may need additional support to be so deeply responsible for delivering what can be a fairly technical and nuanced intervention…”, requiring group management. We speculate that providing greater support for the mentors would have better fulfilled the program design. Misalignment between program design and necessary support for mentors appears to hinder effectiveness. It should be noted that mentor support does not have to take the form of training. Alternatively, it can include having staff support mentors or providing materials that scaffold elements that the program values in the mentoring relationship.

### Optimizing the Role of Mentoring Within Multi-Component Programs

A final theme involves the optimization of mentoring, especially within the context of multi-component programs. The optimization and enhancement of mentoring may require consideration of different approaches to mentoring as well as different modes of connection and communication. It may also require that the mentoring component of a program be clearly delineated from other program components, such that each serves distinct but complementary functions. This theme of optimization also serves as a reminder that too many components that do not align with program aims may dilute the impact of the mentoring component.

In terms of optimization, the combination of different approaches to mentoring (e.g., developmental versus instrumental) emerged as a noteworthy characteristic of effective and promising programs, particularly in relation to Better Futures. One of the main factors highlighted in the Insights as contributing to the program’s large effect sizes across outcomes was that their mentoring was “highly ‘instrumental’…but the way that the program does this is also relationship-driven, and there is a heavy emphasis on personal growth, reflection, and peer support” [i.e., developmental mentoring]. The program supports instrumental mentoring in a flexible way by providing mentors with a menu of different experiences they can pursue relevant to post-secondary transition (e.g., figuring out housing). Mentors can then select the experience that is most relevant to their mentee. Alongside these offerings, Better Futures also attends to fostering personal relationships between young adult mentors and mentees.

The modes of connection and communication utilized by programs are also a consideration in the optimization of mentoring. For instance, the integration of virtual and in-person modes of connection and communication may strengthen programs’ capacity to achieve their desired outcomes. When discussing the E-Mentoring Program for Secondary Students with Learning Disabilities, rated promising, the Insights argue that: “programs that are intended to help a youth through a difficult transition should think about whether online communication could strengthen their program design or outcomes.” In some cases, advancements in technology can shift how programs think about integrating online interactions with a more traditional, in-person program. As stated earlier, modifications should be intentionally administered in ways that align with program theory and that prioritize the particular needs of the youth served.

Optimizing mentoring also involves considering how well other program components complement or support it. Discussions of programs rated as having no effects underscored this point. For instance, the Insights argue that while Chance UK was research-based and well-designed, it “may have had null effects because their target population and goal of behavioral change needed to complement mentoring with behavioral interventions.” In the case of One Summer Plus, an employment program rated as no effects, the Insights propose that the program may have seen no outcome differences because the addition of a staff-led skills curriculum inadvertently weakened the mentoring impact. Instead, mentoring could have been used more intentionally as an organic space for developing skills and applying them in the real world.

In the review, examples arose where programs included too many components that did not align with program aims. At times, multiple components may distract from the larger program aim and dilute the impact of the mentoring component in program evaluation, possibly contributing to a rating of no effects. In these cases, how can evaluations discern the impact of mentoring on its own? Pathways to Education, rated as promising, provides an example of the difficulty of teasing out the role of mentoring in evaluation outcomes. As stated in the Insights, “Clearly, Pathways youth are receiving mentoring, perhaps lots of it, but the details in how that works in synergy with the other program components still remains a bit of a mystery,” especially when “the mentoring that happens seems inadequately described in the study cited in the formal review.” In contrast, the Insights underscore how delineating the mentoring component was clear in the design of Quantum Opportunities, rated as effective, because it gives “mentors clear roles and responsibilities within the broader suite of supports.” Delineating the mentoring component in multi-component programs is a challenging and critical process in evaluating practice.

## Discussion

This study identified themes evident across the Insights for Mentoring Practitioners accompanying youth mentoring program profiles reviewed for effectiveness in the CrimeSolutions repository. Four themes emerged as key to designing and evaluating mentoring programs: (1) ensuring alignment across goals, design, implementation, and evaluation; (2) connecting the intervention to mentees’ home, parents, and larger environment; (3) tailoring mentor engagement and support to effectively serve youth; and (4) optimizing the role of mentoring within multi-component programs. Rather than pointing to a single template for an effective program, the findings suggest leaders of effective and promising programs carefully consider each aspect of the mentoring process in light of their specific goals, youth needs, available resources, and evaluation. This section explores how the themes speak to extant literature, implications for practitioners, and how scrutinizing evaluation designs in and of itself is warranted to carefully measure and assess promising programs.

The strongest theme was the intentional alignment between program goals and implementation, a trend associated with stronger program effects and widely emphasized in mentoring literature. As Luo and Stoeger (2023) note, “an effective mentoring program starts with careful consideration of its objectives and target population” (p. 3073). Similarly, in a model proposed by Cavell et al. (2021), the primary focus is on aligning the form of mentoring with youth needs and strengths. This alignment includes connecting the mentoring approach with program objectives to ensure evaluation is precise and clear in assessing impact. Using a theory of change alongside a logic model may be critical to achieving alignment because a theory of change considers expected change in the context of factors unrelated to the program (Garringer, 2014) and the proximal steps that need to be measured as precursors to distal goals. A logic model can serve as a launching pad for discussion about how program components will achieve alignment and set a foundation for designing evaluations (Caldwell et al., 2018). Given that about a third of programs were rated no effects, and a few rated as effective, there is a strong case for iterative cycles of data-driven strengthening aligned with improvement science principles. Alignment should be revisited regularly, as continuous improvement is key in youth program development (McElvain et al., 2014). A cycle of testing and refining is not only key for program developers but also for funders and policymakers seeking to strengthen the field of mentoring.

In programs that demonstrated alignment, care was taken to determine the optimal role for mentors; recruitment, selection, training, activities, and support were tailored accordingly. Perhaps for this reason, promising and effective programs tended to avoid problems involving a lack of clarity regarding the mentor’s role—the very kind of problem noted among programs rated as no effects. This is supported by Keller et al. (2023)’s study finding that support for program alignment with MENTOR’s *Elements of Effective Practice for Mentoring* was associated with higher levels of mentor satisfaction with the relationship. In other words, alignment and intentionality in program practices translate to higher quality relationships.

Effective or promising programs were noted for deliberately leveraging resources to strengthen mentoring relationships, increasing the likelihood of positive outcomes. In some cases, mentors were encouraged to connect with mentees’ parents and friends, enlisting this larger network in the mentoring process. Some programs partnered mentors with social workers or other professionals to work together to monitor youth, address needs, and enhance outcomes. Varga and Zaff (2018) would describe this as intentionally strengthening a mentee’s “web of support”—the “network of relationships youth have with adults and peers across contexts in which supports are provided that help the young person advance in development” (p. 4).

In more effective, multi-component programs, where mentoring was supplemented with additional services, care was taken to determine how best to incorporate mentoring alongside other components. Such programs prioritized the entire process of building, enhancing, and maintaining high-quality mentoring relationships. This often resulted in intentional plans for developing, maintaining, and reinforcing impactful mentoring relationships—tailored to the context in which a program and mentor operated, such as extra training, and/or involving helping professionals to support outcomes beyond the domain of a volunteer mentor (Rhodes, 2020).

To date, meta-analytic studies show relatively strong effect sizes that tend to be associated with programs that adopt a range of practices designed to enhance mentoring relationships, such as setting clear expectations concerning the relationship, providing ongoing training and support for mentors, and involving parents in the mentoring process (DuBois et al., 2002; Raposa et al., 2019; Rhodes & DuBois, 2008). The themes in our study provide insights into how such practices unfold on the ground in ways that can be meaningful for mentoring program leaders.

### Implications for Practice

Findings could help program leaders identify considerations, decision points, and options for improving or maximizing the effectiveness of their own programs. This can help leaders reflect on whether research they encounter has relevance to them and, if so, how they might integrate new ideas in intentional ways. Questions to spark this reflection include:

- Is the design of the program well-suited to achieving its intended outcomes? This could include developing a theory-based logic model aligned with youth needs and the local context to ensure goodness of fit, as well as naming proximal steps toward distal goals.
- Is the program being implemented in a manner consistent with this design “blueprint?” This might involve training staff and mentors on program theory and expectations to strengthen fidelity to the program model. This may also include understanding what core elements need to be implemented with fidelity, and what elements can be adapted.
- Are evaluation activities focused on outcomes of high priority and relevance in the context of the program’s goals and strategies for achieving them? When acting on evaluation outcomes, is there scrutiny of how the number of outcomes tested may shape findings? This may involve using targeted evaluation activities to support continuous improvement, like focusing on fewer outcomes that align closely with the blueprint.
- In what ways would building bridges between program content and the outside environment in which youth spend significant amounts of time support program goals? Are there people in the youth’s ecology who could be leveraged to supplement mentoring? Who would be most valuable to leverage for the program’s focus and reach?
- In a perfect world, what would the mentors accomplish based on the program focus? What roles and tasks are most aligned with the needs of youth served? What structures can be implemented to reach this ideal? This may involve specifying the program focus to better reinforce mentor roles specific to youth needs and strengths.
- If relevant, how does a program delineate the mentoring component in a multi-component program? This may require reflecting on the intention of incorporating mentoring among the other components. Adding mentoring in and of itself is not inherently good, especially if its role in the intervention or evaluation is poorly positioned.

The NMRC’s website includes resources for technical assistance and training that may be helpful for decision points relevant to these questions at https://nationalmentoringresourcecenter.org/.

Even with all the considerations above, it is important to note that when considering a program’s focus and interpreting a program’s evaluation outcomes, one must recognize the socio-historical context locally and more broadly. For instance, if one is measuring arrests in an environment at the height of stop and frisk, one should interpret changes in that outcome for mentees in that context. Or, if a program is targeting unfavorable outcomes produced by larger inequities in society and institutions, one must consider where the program can intervene, and where it may be hindered because it is working against larger ingrained issues.

Programs must also consider cost relative to impact as they make program decisions. While this consideration did not emerge as key in our analysis, it was raised at several points across the Insights reviewed. Evaluating programs inclusive of cost allows us to intentionally consider alignment of program elements with program goals. It also equips programs to consider the effectiveness of program investments, particularly in light of limited resources, relative to various program goals, components, and outcomes. As needed, programs can pool resources and incorporate multiple disciplines to learn from one another. This can inform future program investments with an eye toward alignment, a theme within this analysis.

Finally, funders and policymakers could use the themes to embrace a test-and-improve approach. The themes suggest innovation should be encouraged as programs work toward considering these themes and engaging in continuous improvement. Every best practice was something that someone was doing “wrong” until they realized what worked better. Clearinghouses can have the unintentional impact of stifling innovation when a program is rated as no effects. Supplementing with program profiles may point to intentional ways programs could improve.

### Limitations and Future Research

The 47 programs in CrimeSolutions may not be representative of the full universe of mentoring programs. Additionally, findings do not address the value of mixed-method evaluations that would enhance the usefulness of this type of commentary. Furthermore, all Insights were written by one person with decades of experience supporting practitioners. This may introduce bias; however, this is the ideal person to engage in this work because of their expertise in translating research into usable information. Future research could explore ways to engage in translational work when supplementing clearinghouses with program profiles. Insights are limited to the available evidence regarding program effectiveness. With notable exceptions, the evaluations that constituted the evidence base did not include analyses that could guide discussion of potentially influential processes and focus on those with the strongest empirical support (e.g., as mediators of effects). Plus, the effectiveness ratings provided by any repository are a function of their stated purposes, and the intricacies of that repository’s review process and framework. In the case of CrimeSolutions, efforts are underway to incorporate ratings specific to different types of outcomes (e.g., promising for one, but not effective for another; LeVigne, 2024). These considerations, while not this study’s focus, are nonetheless germane. Finally, the aim of this study was to generate hypotheses—not definitive conclusions (i.e., hypothesis testing), so findings should be considered in the context of this important caveat and limitation.

## Conclusion

Limitations notwithstanding, these findings speak to the value of periodically taking stock of trends and patterns in the reviews of individual interventions that are the focus of evidence-based program repositories. Furthermore, it is apparent from our analysis that commentaries such as the Insights for Mentoring Practitioners can offer detail and depth on implications for practice that complements the more descriptive and research-oriented content of traditional program profiles. When such commentaries are analyzed collectively, higher-order themes can be elucidated to suggest broader principles for strengthening practice.

## Supplementary Information

Below is the link to the electronic supplementary material.

ESM 1 (PDF 343 KB)

## Data Availability

The Insights for Practice used in the dataset are available on the National Mentoring Resource Center's website under Program Reviews.

## Footnotes

1 Crimesolutions designates “high-quality RCTs” as those rated highly (scores of 2.0 or higher) by both reviewers on Design Quality, which takes into account research design, sample size, use of appropriate statistical adjustments, instrumentation quality, threats to internal validity, length of follow-up period, and the degree to which the study examined for the presence of displacement of crime or diffusion of benefits, if applicable. The direction of the outcome evidence must also align with the program’s overall rating (i.e., effective, promising, or not effective), though none in this report was excluded based on this.

## References

[CR1] Braun, V., & Clarke, V. (2006). Using thematic analysis in psychology. *Qualitative Research in Psychology,**3*(2), 77–101.

[CR2] Caldwell, L. L., Witt, P. A., & Baldwin, C. K. (2018). Intentional programming using logic models. In P. A. Witt & L. L. Caldwell (Eds.) *Youth development principles and practices in out-of-school time settings *(pp. 297–320). Sagamore-Venture.

[CR3] Cavell, T., Spencer, R. & McQuillan, S. (2021). Back to the future: Mentoring as means and end in promoting child mental health. *Journal of Clinical Child & Adolescent Psychology, 50*(1), 1–19. 10.1080/15374416.2021.1875327 33689520

[CR4] Collins, S. E., et al. (2018). Community-based participatory research: Towards equitable involvement of community in psychology research. *American Psychologist, 73*(7). 10.1037/amp0000167 PMC6054913 29355352

[CR5] DuBois, D. L. (2017). Prevention and promotion: Toward an improved framework for research and action. *Handbook of Community Psychology,**1*, 233–251.

[CR6] DuBois, D. L., Holloway, B. E., Valentine, J. C., & Cooper, H. (2002). Effectiveness of mentoring programs for youth: A meta-analytic review. *American Journal of Community Psychology,**30*, 157–197. 12002242 10.1023/A:1014628810714

[CR7] DuBois, D. L., & Karcher, M. J. (2014). Youth mentoring in contemporary perspective. In D. L. DuBois & M. J. Karcher (Eds.), *Handbook of youth mentoring* (2nd ed., pp. 3–13). Sage.

[CR8] Feucht, T. E., & Tyson, J. (2018). Advancing “what works” in justice: Past, present, and future work of federal justice research agencies. *Justice Evaluation Journal,**1*(2), 151–187.

[CR9] Garringer, M. (2014). Does your program have a fully-developed theory of change? *The Chronicle of Evidence-Based Mentoring*. https://www.evidencebasedmentoring.org/poll-does-your-program-have-a-fully-developed-theory-of-change/

[CR10] Garringer, M., McQuillin, S., & McDaniel, H. (2017). Examining youth mentoring services across America: Findings from the 2016 National Mentoring Program Survey. MENTOR.

[CR11] Glynn, D. (2021). Qualitative research methods in translation theory. *SAGE Open*. 10.1177/21582440211040795

[CR12] Hagler, M. A., Jones, K. V., Anderson, A. J., McQuillin, S. D., Weiler, L. M., & Sánchez, B. (2023). Striving for safety, impact, and equity: A critical consideration of AJCP publications on formal youth mentoring programs. *American Journal of Community Psychology,**72*, 258–270. 37807945 10.1002/ajcp.12702 PMC11473108

[CR13] Hseih, H. F., & Shannon, S. (2005). Three approaches to qualitative analysis. *Qualitative Health Research,**15*(9), 1277–1288. 16204405 10.1177/1049732305276687

[CR14] Keller, T. E., Drew, A. L., Herrera, C., Clark-Shim, H., & Spencer, R. (2023). Do program practices matter for mentors?: How implementation of empirically supported program practices is associated with youth mentoring relationship quality. *Journal of Community Psychology,**51*(8), 3194–3215. 36840743 10.1002/jcop.23019

[CR15] LeVigne, N. (2024, Feb 16). Changes coming to CrimeSolutions. Retrieved from https://crimesolutions.ojp.gov/changes-coming-crimesolutions.

[CR16] Luo, L., & Stoeger, H. (2023). Unlocking the transformative power of mentoring for youth development in communities, schools, and talent domains. *Journal of Community Psychology,**51*(8), 3067–3082. 37555757 10.1002/jcop.23082

[CR17] McElvain, C. K., Moroney, D. A., Devaney, E. D., Singer, J. S., & Newman, J. Z. (2014). *Beyond the Bell: A toolkit for creating effective afterschool and expanded learning programs (4th ed.)*. Washington, DC: American Institutes for Research.

[CR18] Padgett, D. (2006). *Qualitative Methods in Social Work Research*. New York, NY: SAGE Sourcebooks for the Human Services.

[CR19] Pluye, P., & Hong, Q. N. (2014). Combining the power of stories and the power of numbers: Mixed methods research and mixed studies reviews. *Annual Review of Public Health, 35*(1), 29–45. 10.1146/annurev-publhealth-032013-182440 24188053

[CR20] Rao, V. & Woolcock, M. (2003). Integrating qualitative and quantitative approaches in program evaluation. *The impact of economic policies on poverty and income distribution: Evaluation techniques and tools*, 165–190.

[CR21] Raposa, E. B., et al. (2019). The effects of youth mentoring programs: A meta-analysis of outcome studies. *Journal of Youth and Adolescence,**48*, 423–443. 30661211 10.1007/s10964-019-00982-8

[CR22] Rhodes, J. E. (2020). *Older and wiser*. Cambridge: Harvard University Press.

[CR23] Rhodes, J. E., & DuBois, D. L. (2008). Mentoring relationships and programs for youth. *Current Directions in Psychological Science,**17*(4), 254–258.

[CR24] Stephenson, R., Cohen, M., Montagnet, C., Bobnis, A., Gies, S., & Yeide, M. (2014). Model Programs Guide Implementation Guides: Background and user perspectives on implementing evidence-based programs. Washington, DC: OJJDP, Office of Justice Programs, U.S. Department of Justice. https://ojjdp.ojp.gov/sites/g/files/xyckuh176/files/media/document/ImplementationGuides.pdf

[CR25] Tebes, J. K. (2005). Community science, philosophy of science, and the practice of research. *American Journal of Community Psychology,**35*(3–4), 213–230. 15909796 10.1007/s10464-005-3399-x

[CR26] Tebes, J. K. (2018). Team science, justice, and the co-production of knowledge. *American Journal of Community Psychology,**62*(1–2), 13–22. 10.1002/ajcp.12252 29882968 10.1002/ajcp.12252

[CR27] Titler, M.G. (2004). Methods in translation science. *Worldviews on Evidence-Based Nursing, 1*(1), 38–48. 10.1111/j.1741-6787.2004.04008.x 17147757

[CR28] Tolan, P. H., Henry, D. B., Schoeny, M. S., Lovegrove, P., & Nichols, E. (2014). Mentoring programs to affect delinquency and associated outcomes of youth at risk: A comprehensive meta-analytic review. *Journal of Experimental Criminology,**10*, 179–206. 25386111 10.1007/s11292-013-9181-4 PMC4224303

[CR29] Varga, S., & Zaff, J. (2018). Webs of support: Integrative framework of relationships, social networks, social support for PYD. *Adolescent Research Review,**3*, 1–11.

