# Supplementary material for: Supplementing Program Profiles in Evidence Clearinghouses with Insights for Practice: a Qualitative Investigation of Application to Youth Mentoring Programs in CrimeSolutions
Source: Prev Sci. 2025 Oct 8;26(7):1087–98. doi: 10.1007/s11121-025-01841-8 (PMC12627147; doi:10.1007/s11121-025-01841-8)
Supplement: Supplementary file 1 — (PDF 343 KB) [file 11121_2025_1841_MOESM1_ESM.pdf]

Supplementary Material/ Online Resources

Supplementing Program Profiles in Evidence Clearinghouses with Insights for Practice: A Qualitative Investigation of Application to Youth Mentoring Programs in CrimeSolutions

Online Resource 1: This is a summary of the programs and evaluations referenced in the articles' data sample, organized by evidence rating.

Summary of Mentoring Programs in Practitioner Insights

Program Name	Program Description
**Programs Rated Effective:**	
Better Futures Program	A program designed to help young people in foster care and with serious mental health challenges prepare for postsecondary education.
Eisenhower Quantum Opportunities	Also known as the Eisenhower Foundation's Quantum Opportunities Program, this program is an intensive, year-round, multicomponent intervention for high-risk minority students from inner-city neighborhoods, which is provided throughout all 4 years of high school.
Great Life Mentoring	A one-on-one mentoring program in which youth ages 7 and up referred from a community mental health agency received support from adult volunteers with whom they spent 2 to 3 hours weekly on positive community activities.
**Programs Rated Promising:**	
Academic Mentoring Program for Educational Development (AMPED)[1]	A school-based program for 6th and 7th graders at risk of academic failure designed to improve academic performance and life satisfaction and to reduce absences and behavioral infractions.
Achievement Mentoring Program (AMP)	An school-based intervention for urban minority freshmen at risk of dropping out of high school designed to enhance school-related cognitions and behaviors.

[1] The AMPED (Promising) program was formerly The Brief Instrumental School-Based Mentoring Program (No Effects). Due to substantial updates to the program AMPED was reviewed as a distinct program.

An E-mentoring Program for Secondary Students with Learning Disabilities	A school-based program that pairs college student volunteer mentors with high school students (10th-12th graders) with mild learning disabilities to help them better identify their postsecondary career goals.
Baloo and You (Germany)	A school-based one-to-one mentoring program that pairs volunteer college student mentors with disadvantaged elementary school children to enrich their social environment and enable their acquisition of new skills.
Bottom Line	A college counseling program that promotes 4-year college enrollment and completion for low-income, first-generation students.
Check & Connect Plus Truancy Board (C&C+TB)	A school-based program that integrates a case-management framework for providing social support to truant youth through one-to-one mentoring by juvenile court probation officers. The goals of the program is to improve school attendance and renew progress toward graduation.
Coaching for Communities (United Kingdom)	A 5-day residential retreat followed by 9 months of one-on-one mentoring by adult community volunteers for mid-to-late teens who display low levels of antisocial behaviors in more than one area but who do not display persistent delinquency
Cognitive–Behavioral Intervention for Children with Emotional and Behavioral Disturbances[3]	A cognitive behavioral one-on-one mentoring intervention delivered by paraprofessionals employed at a local community center designed to improve child behavior and family functioning among 8- to 12-year olds with mental health disorders and their primary caregivers.
Cognitive–Behavioral, Group-Mentoring Intervention for Children with Emotional and Behavioral Disturbances	A cognitive behavioral group mentoring intervention delivered by paraprofessionals employed at a local community center designed to improve child behavior and family functioning among 8- to 12-year olds with mental health disorders and their primary caregivers.
Cross-Age Peer Mentoring Program[2]	A school-based peer mentoring program in which high school students provide one-on-one mentoring to late elementary and early middle school students.
Experience Corps	A one-on-one tutoring and mentoring program delivered by older adult paid mentors (age 55+) to elementary school-aged children (grades 1-3) at risk of academic failure to improve their literacy outcomes.

[2] In three cases, programs had distinct enough features that they were considered as two separate programs for the purpose of review: Challenging Horizons (both no effects), Check and Connect, (One no effects and one promising) and the Cognitive Behavioral Intervention for Children with Emotional and Behavioral Disturbances (both Promising).

Eye to Eye	A group-mentoring afterschool program in which elementary and middle school students with the diagnosis of a learning disability (LD) or attention-deficit hyperactivity disorder (ADHD) meet with high school or college student mentors who also have LD/ADHD, to discuss and address their strengths and challenges.
Fostering Healthy Futures Program	A one-to-one mentoring intervention delivered by graduate students in psychology and sociology designed to improve the well-being of children ages 9-11 recently placed in foster care due to child maltreatment.
Gear Up! Academic Mentoring in Mathematics	A school-based mentoring program for low-income high school students designed to increase academic achievement and college attendance and success.
Helping One Student to Succeed (HOSTS) Program	A structured, one-on-one tutoring and mentoring intervention that was designed to improve language arts skills among low-achieving students in kindergarten through 12th grade.
Home-Visiting Program for Adolescent Mothers	A community-based program in which adolescent mothers met with trained home visitors who delivered a parenting and an adolescent curriculum.
KEEP SAFE	A multicomponent intervention, including one-on-one mentoring with a recent female college graduate, that was designed to prevent delinquency and substance misuse for girls in foster care transitioning from elementary school to middle school.
Mentoring Program for Youth-Headed Households in Rwanda	This is a mentoring program that uses volunteer community members to strengthen the supportive environment and improve psychosocial outcomes among youth living without an adult caregiver in rural Rwanda.
Pathways to Education (Canada)	A multicomponent program that aimed to improve academic outcomes for high school students from low socioeconomic backgrounds through mentorship from a student-parent support worker (program staff).
Peraj Mentoring Program (Mexico)	A mentoring program for fifth- and sixth-grade public school students who are at increased risk for underachievement and antisocial behaviors. The program's goals are to strengthen a students' self-esteem, social skills, motivation, and study skills using college student mentors.
Reading for Life (RFL)	A diversion program in which juveniles ages 13-18 who have committed nonviolent offenses study works of literature and classic virtue theory in small groups led by trained volunteer mentors. The goal is to foster moral development and reduce recidivism.
Rochester Resilience Project (RRP)	A school-based intervention to improve the social-emotional and behavioral skills of young children at risk for mental health disorders and substance abuse.
SAM (Solution, Action, Mentorship) Program for Adolescent Girls	A school-based, substance-use-prevention program for adolescent girls that uses solution-focused brief therapy and community and peer mentorship.

School-Based Mentoring Program for At-Risk Middle School Youth	A program that offered one-to-one mentoring to at-risk students in 7th to 9th grades in an urban middle school setting to reduce their discipline referrals and school absences and to improve their school connectedness.
Sources of Strength	A school-based suicide prevention program designed to build socioecological-protective influences across a full student population using youth opinion leaders from diverse social cliques to develop and deliver messaging aimed at changing the norms and behaviors of their peers.
Youth Advocate Programs, Inc.	An intervention designed to prevent future criminal activity among system-involved youth through using short-term, high-intensity relationships with paid mentors, referred to as Advocates.
Youth-Nominated Support Team-Version II (YST-II)	A program designed to provide adult support to suicidal youth following psychiatric care. Youth nominated a caring adult mentor with whom they already had regular contact.

Programs Rated No Effects:

A Stop Smoking in Schools Trial (ASSIST) Program	A school-wide smoking prevention program designed to spread and sustain norms of non-smoking behavior among 12-13 year olds using influential peer opinion leaders.
Arches Transformative Mentoring Program	A group mentoring program that seeks to reduce recidivism of youth on probation in New York City, using an interactive journaling curriculum based on cognitive-behavioral principles.
Brief Instrumental School-Based Mentoring Program	A school-based one-to-one mentoring program that pairs volunteer undergraduate and graduate student mentors with at-risk middle school students to improve their academic performance, promote school connectedness, and decrease disciplinary actions.
Challenging Horizons Program – After-School Version (CHP-After School) [3]	An after school one-to-one mentoring program that pairs volunteer undergraduate mentors with 10-14 year olds with attention deficit hyperactivity disorder (ADHD) to deliver skills trainings designed to help the youth develop, practice, and generalize academic and social skills.
Challenging Horizons Program – Mentoring Version (CHP-Mentoring)[3]	An school-based one-to-one mentoring program that pairs adult teachers mentors (teachers or school staff) with 10-14 year olds with attention deficit hyperactivity disorder (ADHD) to deliver skills trainings designed to help the youth develop, practice, and generalize academic and social skills.
Chance UK	A one-to-one mentoring program for children ages 5-11 that aims to reduce behavioral and emotional problems in children by developing their self-esteem, self-efficacy, social skills, and future aspirations.
Check & Connect[3]	A school-based, structured mentoring program that pairs truant students with teachers or school staff for one-to-one mentoring to reduce school absences and promote student engagement.

Citizen Schools Extended Learning Time Model	An afterschool program that prepares middle school students for academic and social success.
Early Start to Emancipation Preparation – Tutoring Program	A tutoring intervention designed to improve reading and math skills among 14- to 15-year old youths in foster care who are 1-3 years behind grade level in reading and/or math. The program also aimed to build a mentoring relationship between the youth and their college-student tutor and to provide access to independent living workshops.
iMentor's College Ready Program	A mentoring program for urban high school students is designed to improve college readiness.
My Life Mentoring	A combination 1-1 and group mentoring intervention designed to improve transition outcomes for foster youth ages 16-18 by increasing their self-determination skills.
National Guard ChalleNGe Program	An intensive residential program that provides training and services, including structured one-on-one mentoring, to at-risk youth (ages 16 to 18 years). Youth nominate their own mentor, who is paid for participation.
One Summer Plus Jobs Only (Chicago, Ill.)	A summer jobs program in Chicago, Ill., which seeks to reduce youth violence by providing high-risk students (grades 8-12) with part-time summer employment and access to an adult job mentor.
Peer Group Connection (PGC) Program	A high school transition program that targets 9th-grade students in urban high schools who are at risk of dropping out. The goal is to improve high school graduation rates among participating youths by having junior and senior high school students serve as peer mentors.
Project Arrive	A mentoring program for fifth- and sixth-grade public school students who are at increased risk for underachievement and antisocial behaviors. The program's goals are to strengthen a students' self-esteem, social skills, motivation, and study skills using college student mentors.
Promotor Pathway Program	A program that uses a caring adult, called a Promotor, to provide case management, mentoring, and advocacy for youths.
SOURCE (Student Outreach for College Enrollment) Program	A mentoring program in which high school juniors had regularly scheduled one-on-one contacts with trained college advisors to increase college attendance rates.

Online Resource 2: These are two examples of the full narratives in the Insights for Mentoring Practitioners.

Examples of Insights for Mentoring Practitioners

Program: Better Futures

Note: The National Mentoring Resource Center makes these "Insights for Mentoring Practitioners" available for each program or practice reviewed by our Research Board. Their purpose is to give mentoring professionals additional information and understanding that can help them apply reviews to their own programs. You can read this program's full review on the CrimeSolutions.gov website.

In considering the key takeaways from the research on this program that other mentoring programs can apply to their work, it's useful to reflect on the features and practices that might have influenced its rating as "Effective" (that is, a program that shows definitive evidence of effectiveness)…

Perhaps the first thing that jumps out to readers of the evaluation of Better Futures is the substantial effect sizes that this program has achieved. Effect sizes are, essentially, a measure of just how much change from business-as-usual that an intervention or program has made on participants. In general, mentoring programs as a whole produce an effect size that is considered to be "small" (the 2011 meta-analysis led by NMRC Research Board Chair Dr. David DuBois found an overall effect size of .21). But Better Futures as produced impacts that go far beyond these modest gains, with effect sizes ranging from .74 to 1.75 for just about every outcome the evaluation examined. Only the outcomes of "mental health recovery" and "quality of life" failed to show statistically significant changes for participants compared to a randomly assigned control group of youth. But in every other possible outcome, this program produced impacts that were frequently "large" by the standard definition and downright massive in comparison to most mentoring interventions.

So how did Better Futures achieve all this? Let's look at a few meaningful factors:

- ***A great application of relevant theory when designing the program*** – As we've noted in considering other program reviews for the NMRC, one of the keys to success for mentoring programs may be making sure that the services are building on relevant theories and prior research that indicates that mentors will produce results within the context of the youth needs being addressed. Better Futures does an excellent job of this in its design by building on the concepts of Self-Determination Theory, which is premised on the idea that an individual's well-being is influenced by the degree of autonomy and skill he or she has in his or her own self-care. This program clearly is built around the idea of empowering youth in foster care—youth who by nature of their experience in the child welfare system may have felt very, very disempowered in the course their life has taken. The program is built on a foundation of self-determination and that idea that if these youth are put in the driver's seat of their postsecondary planning, and provided with just enough mentoring, skill development, and instrumental support as scaffolding, that the results will be meaningful. And this evaluation certainly seems to indicate that they were correct. Participants empowered in this manner were found to leave this program in a very different place than many of their non-participant peers and all of that arguably starts with the theory of self-determinism and the cultivation of mentoring services that will enhance that self-determinism in mentees.

- ***Normalizing the experience of aging out of foster care and going into post-secondary life*** – Another relevant idea that we can see play out in the design of this program is that of "normalizing" what is essentially an abnormal experience. Youth in the child welfare system live, by default, lives that are anything but normal. The uncertainty, shifting environments, and inconsistent care offered by that system can leave youth feeling like they are very much anomalies among their peers. In fact, the NMRC has reviewed another promising mentoring program for serving foster youth that emphasizes making the experience seem more "normal" by having them interact with peers who are in similar circumstances. Better Futures seeks to achieve this "normalization" by bringing participants together for a multi-day "institute" on a college campus where they can interact with dozens of youth who are facing the same challenges and uncertainties as they head into young adulthood and plan for life after high school. The potential power of bringing together cohorts of youth going through the same difficult transition cannot be overstated as this offers the opportunity for connections to multiple mentors and a wealth of peer learning and information sharing, along with the overarching relief of knowing "I'm not in this alone."

- ***Offering a blend of developmental and instrumental mentoring*** – The support offered by Better Futures is, in many cases, highly "instrumental" in nature—that is, it emphasizes teaching, information sharing, and helping youth achieve some concrete goal or complete some discrete task. In fact, the mentors in the program have a suite of 17 different "experiences" that they are supposed to provide to youth, as well as 11 targeted self-determination "skills" they are supposed to teach, all in the service of preparing a plan for post-secondary education. So the experience of being a mentee in the program appears largely to be that of getting some concrete help on all this planning and completing the often mundane tasks associated with going to college, such as applying for financial aid or figuring out housing circumstances. But the way that the program does this is also relationship-driven and there is a heavy emphasis on personal growth, reflection, and peer support. This is a program that values relationships and encourages participants to, for the first time in their lives, take control of their future and the trajectory of the years to come. This combination of developmental mentoring and instrumental support is very much in alignment with recent theory about how mentors might work best with youth, helping them grow as people generally while also providing targeted instrumental support to overcome hurdles as needed. (For more on this idea, see this special issue of *New Directions for Youth Development*; the full text articles may be available through the services of public or university libraries.)

- ***Picking the right mentors to help "normalize" this transition –*** Another key factor in the success of Better Futures may be who they ask to fill the mentor role. The mentors in this program are all young adults who have been to college and who *also* themselves have been in the foster care system or dealt with mental health issues. One can imagine that seeing a slightly older person who comes from a similar background and has managed to make this transition is highly motivating and empowering to participants. These mentors are likely to have invaluable first-hand experience with the struggles, the stumbling blocks, and the "tips" that can make this transition go well for mentees. It's hard to imagine any old volunteer bringing the same level of understanding, skill, and personal lived experience to the table, no matter how good the training provided. This program does a nice job of backing up its self-determination message with living examples of what that transition looks like on the other side.

- ***Tracking fidelity of implementation… flexibly*** – Another interesting feature of the program is found in those 17 “experiences” and 11 skills that the program tries to give mentees. As suggested earlier in this discussion, when a program is this grounded in meaningful theory and the expected changes, one might assume that fidelity to the model (how rigorously the program does certain tasks) is paramount, even though each mentee is a unique person with unique challenges and needs. But Better Futures finds a very nice middle ground between the rigidity of the model and the flexibility to meet clients where they are at. The mentors in the program are not expected to deliver all 17 of those “experiences” to every mentee, but rather, to select the ones that are most relevant to each of the individual participants. This way, the program can deliver a whole host of relevant skill-building and instrumental supports to every youth in the program, while also maintaining the flexibility needed to customize the experience in accordance with that overarching self-determination principle.

  The mentors are encouraged to provide as many of the experiences as possible, but this can, as noted, be customized and tailored to the individual. The mentors are also responsible for tracking the components of the intervention that each mentee receives. Even though the program allows for a lot of flexibility, their fidelity results speak for themselves: 100% participation in the Summer Institute elements, 99.3% in exposure to the 11 self-determination skills, and 90.4% participation in the 17 experiential activities. These results speak to a program that allows mentors to give mentees the right developmental experiences but also ensure that everyone in the program is getting a robust intervention. After many years in the child welfare system, one can only imagine how nice it must feel to mentees to have services customized to their needs and not be shoved into a one-size-fits-all approach.

In spite of these strong outcomes and innovative approaches, there are some caveats that service providers should keep in mind when thinking about this program:

- The evaluation does not provide a cost-per-match estimate, but one imagines that the multi-day Institute and customized support after are not low-cost. This program is providing a lot of support, but it is unclear how scalable this model is, both for cost and logistical reasons.

- The evaluation also does not examine whether these youth actually went to college more than control youth. One might assume that they did, given their massive gains in measures of planning and preparation, but it’s unclear whether the program actually led to more of these youth attending, and subsequently graduating, from their chosen higher education institutions. A follow-up study looking at those outcomes would be a nice addition to our understanding here.

For more information on research-informed program practices and tools for implementation, be sure to consult the Elements of Effective Practice for Mentoring™ *and the “*Resources for Mentoring Programs*” section of the NMRC site.*

Program: Home Visiting Program for Adolescent Mothers

Note: The National Mentoring Resource Center makes these "Insights for Mentoring Practitioners" available for each program or practice reviewed by our Research Board. Their purpose is to give mentoring professionals additional information and understanding that can help them apply reviews to their own programs. You can read this program's full review on the CrimeSolutions.gov website.

In considering the key takeaways from the research on this program that other mentoring programs can apply to their work, it's useful to reflect on the features and practices that might have influenced its rating as "Promising" (that is, a program that shows s**ome** evidence that it achieves justice-related goals when implemented with fidelity).

- ***Getting the most out of a handful of volunteers.*** Perhaps the most striking thing about the Home Visiting Program for Adolescent Mothers model is just how much effort this program got from its three volunteer mentors. In the version of the program studied by Barnet and colleagues, three volunteer mentors (all African American women from the community being served) met biweekly with a caseload of 10-15 pregnant and parenting teens in the first year of their children's lives, as well as meeting monthly with up to 10 mothers whose children were between 1 and 2 years of age. That's a caseload of as many as 25 "mentees" needing individual meeting times each month! And this was not easy work, either. The study mentions that for several of these parents, meetings needed to be discreetly scheduled outside of the home because of issues around violence and safety in the home. Plus, each meeting needed to use a structured curriculum that offered all participants the same messages and lessons.

  It is unclear from the study whether these volunteers received any kind of stipend, or even small things like reimbursement for mileage driving to all these meetings. But whatever the incentives, these volunteers stuck with the program: Only one of the three mentors left during the four year window the program was studied. This allowed for deep relationships to form between these mentors and their mentees, something the authors hypothesize was likely critical to getting these young mothers to change their attitudes and behaviors around parenting and their own access to healthcare. So while it remains a bit of a mystery as to how the program achieved this remarkable volume and duration of work from their volunteers, the program does serve as a nice example of how programs can really lean on motivated volunteers, especially those who have experience and deep commitment to the community being served.

- ***Integration with social workers and other professionals can be critical to achieving certain outcomes.*** While these strong volunteer mentors served as the face of the program and the main deliverers of the curriculum and intervention, they did not go into this alone. Each mentor also had access to a social worker who was available to make referrals to other services and monitor issues related to safety and mental health. This "backup" from experienced professionals meant that the mentors could focus more on relationship building and emphasizing the messages in the curriculum, knowing that they had another staff member to lean on if the parent was experiencing a more critical situation in the home.

  However, the coordination of services with health care professionals proved more challenging. The evaluation noted that the mentored parents did not connect with primary care physicians any more than the "control" group did and that the lack of communication between mentors and primary care physicians may have meant that some of the participants' needs around

depression, contraception, and other health concerns went unmet. Of course, sharing information with health care professionals is challenging for any community based program due to both logistical and privacy concerns. Mentoring programs should think carefully about how and when their services can interface with doctors and other medical professionals. It may be challenging to achieve program outcomes that are largely dependent on coordination and communication that may prove elusive.

- ***The need to tap into mentee motivations.*** One of the minor points in the evaluation write up is worth examining more closely here. The authors note that one of the possible reasons that the program failed to influence contraception use and avoidance of second pregnancies is that the curriculum was great at providing information *about* contraception options and encouraging access to doctors who could prescribe them (at least the hormonal ones), but was not great at tapping into the motivations of mentees that would get them to be proactive about their birth control. They point out that other studies of effective birth control programs often highlight the importance of helping youth identify motivating factors that would spur their contraceptive use. This might be a goal for the future, a connection to their own sense of self, or even just a desire to not repeat what they consider to be a mistake. But the curriculum in use here emphasized information and health care access over motivations to use those things in practice. This serves as a good reminder to mentoring programs that you can provide your mentors with all kinds of information and tools, but if you are not motivating mentees to take advantage of them, it can often be for naught. Helping youth tap into their sense of purpose and goals likely can go a long ways towards making sure they take full advantage of what the program provides. Evidence of this can be seen in other programs reviewed on this site that emphasize motivation and self-empowerment.

- ***What about the next generation?*** While this program, and the evaluation cited in this review, were both focused on outcomes for the teen mothers being served, one also has to wonder about the impact of these services on the infants in their care. Surprisingly, the study did not include any information about whether these moms were more diligent about pediatric check-ups, indicators of better parent skills in practice, or whether their children were healthier or better cared for than those of moms in the control group. While this program was intensely targeted at parents, the reality is that those intended outcomes were in service of their children, hoping to ensure that these infants got the love and support they needed during the critical early years. Hopefully future evaluations of this model—and others intended to support young parents, such as the Office of Juvenile Justice and Delinquency Prevention's Second Chance Act Strengthening Relationships Between Young Fathers, Young Mothers, and Their Children initiative—will examine whether the outcomes exhibited by the moms (and dads!) in turn translate into improved early childhood outcomes for their children. That next generation is where the community-level impact of a program like this would be truly felt.

For more information on research-informed program practices and tools for implementation, be sure to consult the Elements of Effective Practice for Mentoring™ *and the "Resources for Mentoring Programs" section of the NMRC site.*

Program: Check & Connect

Note: The National Mentoring Resource Center makes these "Insights for Mentoring Practitioners" available for each program or practice reviewed by our Research Board. Their purpose is to give mentoring professionals additional information and understanding that can help them apply reviews to their own programs. You can read this program's full review on the CrimeSolutions.gov website.

In considering the key takeaways from the research on this program that other mentoring programs can apply to their work, it's useful to reflect on the features and practices that might have influenced its rating as "No effects" (that is, a program that has strong evidence that it did not achieve justice-related goals).

1. Flexibility in implementation can be a blessing and, perhaps, a curse.

One of the real conundrums mentoring programs face is how they can both build on and implement research-based "effective" practices and program models while also allowing for enough flexibility to customize an intervention or specific practice for local context or needs. It can be a challenge to take something that worked in one place and apply it to a new population of youth, a new city or school, or to vary up aspects of the work to match the availability of local resources. There is a whole body of research devoted to these questions of implementation science: What components of a program are critical to keep as is and which can be tweaked or even discarded? Did something that worked well for one group of kids work for a different one? Are there contextual factors that doom some efforts before they start? When is a model no longer *a model*?

Check & Connect is a program that has been thoughtfully developed and that has garnered considerable interests from education institutions and nonprofits around the country looking for a solution to issues of school truancy and disengagement. Both of the evaluation reports discussed in the Crime Solutions review mention the many previous implementations of Check & Connect around the country, reviewing both the findings of those prior efforts and the role those findings played in the decision to implement that specific model in these new settings.

But what is striking about the two implementations of Check & Connect in this review is how different they are, despite being essentially the same program model. The study by Guryan and colleagues (Guryan, et al., 2017) focuses on a test of the program serving youth grades K-8. The average student in their cohorts was around eight and a half years old, likely a 3rd grader. The other study, Heppen et al., 2017) tested Check & Connect with students starting in grade 10, meaning that they received support from their mentor in their sophomore and junior years of high school. Paradoxically, the authors of the Heppen study conclude that the Check & Connect model probably works best for younger students that are not already so credit-deficient in 10th grade while the Guryan study was pretty clear that the strongest outcomes were for the older students who participated in 7th and 8th grade. It was far less impactful for younger elementary students. This does not mean that these two studies together have inadvertently found that the sweet spot for this program model is middle school students, but it does highlight just how much results can differ from one program model across the ages of youth served.

The differences in implementation across the two studies goes deeper than just the ages of the youth. In the Guryan study, parent engagement is described as one of the four cornerstones of the Check & Connect intervention and, indeed, mentors contacted parents or guardians and average of twice a month. In the Heppen study, the role of parents is scarcely mentioned and their engagement or contact with mentors isn't reported–their role seems limited to being involved in more intensive interventions "when necessary" for some students (p.4). The mentors in Guryan were employees of a community-based nonprofit hired to work in the schools, whereas in Heppen they appear to have been district

employees tasked with serving students in this role. In one study, mentors has a caseload of 30 students, in the other they typically had 50-60–double the number of students.

And although both evaluation reports note that referrals to other services (e.g. mental health providers, dedicated tutoring, or broader wraparound services for the family) are absolutely critical for the intervention to succeed, neither report notes the volume or nature of those referrals or their impact on the outcomes studied. The authors do note that earlier implementations of Check & Connect focused on students with physical or learning disabilities, so perhaps those referrals were more important in those contexts. But still there is little in these reports about how much mentors when beyond themselves in offering help to youth and families.

Both reports here note in that, in each instance, Check & Connect was implemented with fidelity, as intended. And both programs had mentors go through the recommended training and used the manuals offered by the developers. But in reading these two evaluation reports, one can't help but wonder if these two programs were so dissimilar as to leave the question of what this model truly looks like at peak implementation, or who it serves best, up for some debate. This may be a case where the general flexibility of the intervention may encourage its application in contexts where it will be more challenging or when another approach might be more effective. Sometimes rigidity of implementation may be the practitioner's best friend.

2. How much time together is needed for a relationship to be meaningful and "close"?

One of the most intriguing aspects of the Check & Connect model is that it last for two full years of school for participating students. That's a long time in the context of most school-based mentoring programs and it also crosses multiple school years, something that can be a challenge for in-school mentoring programs but that research suggests might be critical for sustaining impacts. Twenty-four months of a mentor at school checking in with you and helping problem-solve issues at school and home sure sounds like it would allow for some real bonding and trust-building between mentor and mentee and plenty of depth to their interactions. In fact, the descriptions of the role of mentors in Check & Connect in the studies discussed in this review describe the meaningful social capital that these mentors provide and the critical role that meaningful relationships play in helping students overcome barriers to success in a socialized context like a school.

But even at two years per relationship, there are questions about how much mentoring happened in these implementations of Check & Connect. This has been a topic of debate in the mentoring universe for some time and the studies presented in this review do little to quell that conversation. In the Guryan study, mentors met with students five times a month on average, although some of that was group meetings with other students the mentor was working with. They also noted that the level of engagement varied quite a bit from mentor to mentor. Each contact with students was described as brief and intended to provide a "nudge." In Heppen, mentors met with their students 37 minutes a month in Year One, 50 minutes a month in Year 2, and 61 minutes a month in Year 3, with around 20 minutes a month over the summers across the two years. Given what most mentoring programs offer, that is very light interpersonal contact–MENTOR's 2016 National Mentoring Program Survey found that approximately 77% of the nation's programs offer youth at least 90 minutes of mentoring a month (Garringer, M., McQuillin, S., & McDaniel, H., 2017). These mentors simply weren't spending very much time with their mentees. And they were split for time across 50 to 60 students! That sounds like a situation where every student was getting that "check" but one wonders if these relationships brought the social capital, personal touch, and authenticity of interaction that is assumed in the model itself. One

wonders if these relationships were… relationships. Other research has hinted that even in-school mentoring relationships need some opportunities to experience fun and playful one-on-one interactions that support bonding and closeness–this has also proven true in other out-of-school mentoring interventions for youth who are struggling with challenging issues. It doesn't seem clear, from the two implementations of the model provided here, that Check & Connect is allowing for that. This is an issue that has been noted for other narrowly-focused school-based mentoring interventions, but there is also evidence that is fixable when programs place a bit more emphasis on that relational bonding. It's not clear how that might happen with a caseload of 50+ high school juniors.

3. When designing an intervention for youth with attendance issues, plan for their inherent mobility.

One of the frustrating aspects of these two evaluations of Check & Connect is that the mobility of the students themselves influenced the delivery of services and the ability to achieve the stated goals of the programs. The Heppen study, in particular suffered from students moving within and out of the district. Those students had much worse outcomes than students who stayed either at the same school or in the district. While there are many practical reasons why mentors would have a very hard time continuing to follow up with students who moved out of the district, it is also true that the developers of these programs had plenty of knowledge that this would be an issue. If the goal was to continue to offer the intervention as intended to these students once they enrolled, one wishes that they had a better contingency plan–dedicated staffing for students who moved out, increased contact with parents when students moved, some ability to travel in-person to neighboring districts, e-mentoring platforms where the relationships could continue–to serve students more effectively. The authors in Heppen conclude that

> *"for highly mobile students, large caseloads may prevent mentors from being able to track down and spend an adequate amount of time working with all of their students… Mentors in this study attempted to connect with students who left district schools, but they found this process difficult and time consuming and were concerned that it was taking time away from other students on their caseload. In response, the program developers and implementation team decided during the study to prioritize delivery to the nontransfer students."*

In other words, they seem not to have prepared adequately for the mobility of a group of students that by definition tends to have high mobility and, as a result, shifted gears on these students mid-study. Practitioners designing services intended to send students on a path towards course completion and graduation like this may want to prepare for that more in the future and draw clearer lines about how those students will and won't be served.

4. Think about how your program can access outcome data at multiple layers and long after services have ended.

The Heppen evaluation offers a nice example of how a school-affiliated program that wants to increase school completion and graduation can track those outcomes even if they are set to occur many years after youth have participated in the program. The evaluators made sure to secure access to school district records to see if youth who participated as freshmen, sophomores, and juniors eventually graduated high school or would up getting a diploma even years later if they happened to leave school prematurely. But as noted above, their study had quite a bit of attrition as youth moved across schools and even out of the district altogether.

To address those circumstances, they went up a layer in the education data system, accessing student records at the state level. Many states have become particularly skilled in collection and sharing student data for research purposes through statewide longitudinal data systems. Programs and evaluators are encouraged to learn what these systems in their areas can provide and use that information to help determine the true impact of programs over time and when students move away (but are still within the state).

For more information on research-informed program practices and tools for implementation, be sure to consult the Elements of Effective Practice for Mentoring™ *and the "Resources for Mentoring Programs" section of the NMRC site.*
